# Mouse model of ocular hypertension with retinal ganglion cell degeneration

**DOI:** 10.1371/journal.pone.0208713

**Published:** 2019-01-14

**Authors:** Ryo Mukai, Dong Ho Park, Yoko Okunuki, Eiichi Hasegawa, Garrett Klokman, Clifford B. Kim, Anitha Krishnan, Meredith Gregory-Ksander, Deeba Husain, Joan W. Miller, Kip M. Connor

**Affiliations:** 1 Angiogenesis Laboratory, Department of Ophthalmology, Massachusetts Eye & Ear Infirmary, Harvard Medical School, Boston, Massachusetts, United States of America; 2 Department of Ophthalmology, Graduate School of Medical Sciences, Gunma University, Maebashi, Gunma, Japan; 3 Department of Ophthalmology, School of Medicine, Kyungpook National University, Kyungpook National University Hospital, Jung-gu, Daegu, South Korea; 4 Department of Ophthalmology, Graduate School of Medical Sciences, Kyushu University, Fukuoka-city, Fukuoka, Japan; 5 Schepens Eye Research Institute of Massachusetts Eye and Ear Infirmary, Boston, Massachusetts, United States of America; Instituto Murciano de Investigacion y Desarrollo Agrario y Alimentario, SPAIN

## Abstract

**Objectives:**

Ocular hypertension is a primary risk factor for glaucoma and results in retinal ganglion cell (RGC) degeneration. Current animal models of glaucoma lack severe RGC cell death as seen in glaucoma, making assessment of physiological mediators of cell death difficult. We developed a modified mouse model of ocular hypertension whereby long-lasting elevation of intraocular pressure (IOP) is achieved, resulting in significant reproducible damage to RGCs.

**Results:**

In this model, microbeads are mixed with hyaluronic acid and injected into the anterior chamber of C57BL/6J mice. The hyaluronic acid allows for a gradual release of microbeads, resulting in sustained blockage of Schlemm’s canal. IOP elevation was bimodal during the course of the model’s progression. The first peak occurred 1 hours after beads injection, with an IOP value of 44.69 ± 6.00 mmHg, and the second peak occurred 6–12 days post-induction, with an IOP value of 34.91 ± 5.21 mmHg. RGC damage was most severe in the peripheral retina, with a loss of 64.1% compared to that of untreated eyes, while the midperiphery exhibited a 32.4% loss, 4 weeks following disease induction.

**Conclusions:**

These results suggest that sustained IOP elevation causes more RGC damage in the periphery than in the midperiphery of the retina. This model yields significant and reproducible RGC degeneration.

## Introduction

Glaucoma is one of the leading causes of blindness worldwide [1, 2] with an estimated 60 million people suffering from the disease [2]. The essential pathogenesis of glaucoma is the loss of retinal ganglion cells (RGCs), accompanied by glaucomatous optic disc neuropathy[3]. Extensive research has identified a number of insults leading to RGC death, including: nitrogen oxide [4], glutamate [5], circulatory disturbances [6], hypoxia [7], mitochondrial dysfunction [8], oxidative stress[9], protein misfolding [10], and elevation of intra-ocular pressure (IOP) [11]. Clinically, IOP lowering agents are the only treatment shown to have a preservative effect on the visual field in patients with glaucoma [12] [13], indicating that elevation of IOP is strongly associated with RGC death in glaucoma [14]. However, the mechanism by which elevated IOP leads to RGC death remains unclear.

To date, there are a handful of established animal models of glaucoma, including genetic models as well as a few models of induced ocular hypertension, which have each significantly expanded our understanding of the molecular and cellular mechanisms involved in the disease. Among the animal models of inherited glaucoma, DBA/2J mice [15–17] are frequently used in glaucoma research; however, the model is slow in developing sustained damage, with severe damage usually observed around 9 months of age [3, 18]. Artificial models in rodents, rabbits, and primates have also been utilized in numerous investigations, including the laser-induced glaucoma model [19, 20], the microbead occlusion glaucoma model [21, 22] the scleral cauterization glaucoma model [23, 24], and the optic nerve axotomy model [25] [26]. Microbead occlusion models have previously demonstrated the ability to induce IOP elevation and subsequent both RGC and their axonal damage. Furthermore, the microbead methodology for this model is not complicated, the model does not induce severe inflammation, and the anterior chamber remains clear during the follow up period [21, 22]. Despite these advantages, the duration of increased IOP is short and the extent of RGC and axonal damage are limited. In order to induce sustained IOP elevation with significant damage to RGCs, we modified the microbead occlusion model in C57BL/6J mice using glue. This enhanced modified model also allows us to take advantage of well-established genetic manipulation platforms (e.g. knock-out strains) in mice with the added benefit of sustained IOP and a corresponding increase in RGC death. In this study, we also describe the pattern of RGC damage in whole retina in detail and subsequent changes in vascular and anterior chamber system accompanied with ocular hypertension (OH).

## Materials and methods

### Animals

6–8 week-old male C57BL/6J mice (The Jackson Laboratory, Bar Harbor, ME) were used in this study. All animal procedures in this study adhered to the Association for Research in Vision and Ophthalmology Statement for the Use of Animals in Ophthalmic and Vision Research and the NIH Guide for the Care and Use of Laboratory Animals, and was approved by The Animal Care and Use Committee of Massachusetts Eye and Ear Infirmary.

### Surgical induction of ocular hypertension

Modifications were made to a well-established microbead occlusion model (Original Method group) [21, 27–29] to induce ocular hypertension. Mice were anesthetized by intraperitoneal injection of a mixture of ketamine (80 mg/kg; Phoenix Scientific, Inc., St. Joseph, MO) and xylazine (16 mg/kg; Phoenix Pharmaceutical, Inc., St. Joseph, MO). After pupil dilation with 5% phenylephrine, 0.5% tropicamide, and anesthetic drops (0.5% proparacaine hydrochloride; Bausch & Lomb, Tampa, FL.), a corneal incision was made using a 31G needle from the temporal to superior cornea. Counterforce of the cornea, with the forceps gripping the limbal area will aid in the cautious manipulation of the 31G needle. Once the tip of the 31G needle penetrates the full-thickness of the cornea, the needle tip should be withdrawn to minimize the inlet size of the cornea incision, which will help to avoid leakage. A glass micropipette (outer diameter/inner diameter; 1.0/0.75 mm) attached to a Hamilton 25μl syringe by an 0.86 mm polyethylene tube and 20G needle was inserted into the anterior chamber via the incision site to inject different compositions of microbead suspensions. Injection should start after placing the tip in the counterpart of the anterior chamber which will help to replace the anterior chamber sufficiently with HA and the beads suspension. Preparation of the microbead suspension for the conventional method (i.e., Original Method group) was conducted as follows. 10 ml stock suspension of FluoSpheres Polystyrene Microspheres (15 μm diameter; orange fluorescence; Life Technologies) was evenly divided into ten tubes (1 ml aliquots) that were left overnight to allow the suspension to segregate. Afterwards, the supernatant was discarded and the beads were resuspended in 50 μl of saline solution. 3.0 μl of the beads and saline suspension (2.0 x 10^7^ beads/ml, as in the original protocol) was drawn into specially made glass pipettes and injected into the anterior chamber as described above.

Our first modification to the conventional (Original Method) protocol involved altering the nature of the beads suspension, in which hyaluronic acid (HA, Provisc; Alcon Laboratories (S.A.) (Pty) Ltd.) was used to suspend microbeads instead of saline solution. To identify the optimal injection volume of the beads suspension in HA, IOP was measured (as described below) for 4 weeks after injecting different volumes of microbead suspensions in HA (ranging from 3.5 μl to 6 μl). Based on the IOP profiles (**S1 Fig**), an injected volume of 6 μl of beads in HA was deemed optimal for comparisons with the conventional protocol (3 μl of beads in saline, 2.0 x 10^7^ beads/ml) and with injections of HA alone (control group). Preparation of the 6 μl suspension of beads in HA (HA+Beads group) was conducted in a similar fashion as with the conventional protocol. Briefly, 10 ml stock suspension of FluoSpheres Polystyrene Microspheres was evenly divided into 1 ml aliquots that were left overnight to allow the suspension to segregate. Afterwards, the supernatant was discarded and the beads were gently mixed in 50 μl of HA for each tube, resulting in a reformulated concentration of 2.0×10^7^ beads/ml of HA. 6 μl of this mixture was then drawn into specially made glass pipettes and gently injected (approximately 1.2×10^5^ microbeads/eye) as described above.

Another modification to the experimental model involved how to seal the incision site after injection. Unlike the conventional protocol [21, 27–29], which uses air to seal the incision site, our new protocol utilized glue (WEBGLUE; Patterson Veterinary Supply, Inc., MA) (**S2 Fig**) to seal the incision site after replacement of the total anterior chamber volume with the HA and beads suspension (HA+Beads group). The glue contained purified n-butyl cyanoacrylate as it allows for rapid and strong adhesion between tissues even in a wet environment such as the tear film covered corneal surface. Glue was also used to seal the incision site for the control group (HA Alone) but was not used in the Original Method group. The glue should be applied evenly such that the surface of the glue on the cornea incision site is flat. The sealing of the cornea incision by glue should be monitored at 1, 6, and 12 hours following the procedure utilizing a microscope while monitoring the IOP at the same time. Whenever the glue patch peeled off, glue repatching was performed in the same manner as the initial application.

### IOP measurement

IOP was measured under general anesthesia with isoflurane inhalation (1.5–2%; Piramal Critical Care Inc.). Measurements were taken within 2–3 minutes after animals were anesthetized, which was determined based on failure to respond to both toe pinch and blink reflex tests. IOP was measured with a TonoLab tonometer (Colonial Medical Supply, Franconia, NH). Anesthetized mice were placed on a platform, and the tip of the probe was placed on a central area of the mouse cornea. Baseline IOP was measured one day before the injection. To assess post-injection IOP elevation, IOP was measured at the following time points: immediately after, 1 hour, 6 hours, 12 hours, and then every 3 days until 30 days post-injection for each experimental condition.

### Histological procedures for angle analysis

For qualitative analysis of the anterior chamber, we analyzed whole eye cryosections 4 weeks after injection. Mice were deeply anesthetized by over dose of ketamine and xylazine and cervical dislocation. Eyes were embedded in optimal cutting temperature (OCT) compound (4583; Tissue Tek; Saukra Finetek, Torrance, CA, USA) immediately after enucleation and frozen by submersing in isopropanol chilled on dry ice.- 10 μm cryosections were cut where beads occluded the angle and stained with Hematoxylin and Eosin (HE).

### Immunohistochemistry

Eyes were placed into fixative consisting of 4% paraformaldehyde (PFA) in PBS for 1 hour, at room temperature, immediately after enucleation. The whole retinas were isolated and placed in blocking buffer (PBS containing 2% goat serum, 2% donkey serum, 3% BSA, and 2% Triton) for 1 hour at room temperature, and subsequently incubated with goat anti-Brn3a polyclonal antibody (Santa Cruz Biotechnology,sc-31984, 1:200) in blocking buffer overnight at 4°C on a vacillating platform. Following this, retinas were washed 3 times for 30 min in PBS containing 0.3% Triton and incubated with the secondary antibody Alexa Fluor 488 (Alexa Fluor 488 conjugated donkey anti-goat IgG, 1:500) for 2 hours at room temperature. For Tuj-1 staining, retinas were incubated with rabbit anti-beta Ⅲ Tubulin polyclonal antibody (Abcam:18207, 1:300) in blocking buffer overnight at 4°C, washed 3 times for 30 min in PBS containing 2% Triton, and incubated with Alexa Fluor 488 (Alexa Fluor 488 conjugated donkey anti-rabbit IgG, 1:500) for 2 hours at room temperature. For staining of the retinal vasculature, retinas were incubated with Isolectin GS-IB4 (Alexa Fluor 647, 1:100) in blocking buffer overnight at 4°C. Prior to flat-mounting (described below), the retinas were again washed 3 times for 30 minutes in PBS.

### Retinal flat-mounts and image acquisition

Retinas were flat-mounted and photographed as previously described [30]. Briefly, whole retinas were carefully resected into 4 petal-shaped lobes and flat-mounted with glass coverslips and mounting medium (Thermo Scientific-Invitrogen, TA-030-FM). A fluorescence microscope (Axio Observer Z1; Zeiss Microscope, Jena, Germany), equipped with a computer-driven motorized stage controlled by image analysis software (Zen pro 2012, Zeiss Microscope, Jena, Germany), was used to capture tiled images (5x objective) of the retinal flatmounts that were stitched together with the aforementioned imaging software.

Images for RGC quantification were obtained using confocal microscopy (TCS SP5; Leica microscope, IL, USA) with the 40x objective. This objective was used to capture the 4 midperipheral and peripheral areas of a whole retina in a single frame (**S3 Fig**). The 4 midperipheral areas were selected in each lobe located within 500 μm of the optic disc. The 4 peripheral areas were selected in the center of each peripheral lobe (**S3 Fig**). RGCs were then counted as detailed below.

### Visualization of RGC density (S4 Fig)

The total number of RGCs was obtained by immunostaining the retinas with Brn-3a, 4 weeks after injection. Using the software Image J, RGCs were manually marked with dots on the retinal photomontages. Next, the dots were automatically quantified and their retinal position extracted using Image J’s “Find Maxima” processing function. RGC distribution was determined by producing nearest-neighbor maps in Image J. We fixed the radius of study (0.165mm) and imported the previously obtained spreadsheet. Those cells within the fixed radius were counted as neighbors. Spatial information was used to plot every RGC, and each RGC was colored using a scale from blue (corresponding to 0 neighboring RGCs) to red (corresponding to 280 or more neighboring RGCs).

### Quantification of axon loss with PPD stain

Mouse optic nerves were dissected 1 mm posterior to the globe and fixed with half-strength Karnovsky’s fixative (2% of paraformaldehyde and 2.5% of glutaraldehyde in 0.1 mol/L sodium cacodylate buffer, pH 7.4;Electron Microscopy Sciences) overnight at 4°C. After fixation, tissue was then rinsed in 0.1 mol/L sodium cacodylate buffer and post-fixed with 2% osmium tetroxide. The tissue was dehydrated in a graded ethanol series and embedded in tEPON-812 resin (Tousimis, Rockville, MD). Semithin sections were cut at 1 μm. The semithin sections were then stained with an aqueous solution of 2% Paraphenylene diamine (PPD; MP Biomedicals) for 30 minutes at RT, rinsed, air dried, and then mounted and cover slipped. Optic nerve cross-sections were imaged at 100x magnification on a Nikon microscope (Eclipse E800, Nikon, Japan) with the DPController software (Olympus, Japan) and 8–10 non-overlapping photomicrographs were taken to cover the entire area of each optic nerve cross-section. Using Image J (Version 2.0.0-rc-65/1.51u), a 50 μM x 50 μM square was placed on each 100x image and all axons within the square (area equal to 0.0025mm^2^) were counted using the threshold and analyze particles function in image J. The average axon counts in the 8–10 images was used to calculate the axon density (axons/mm^2^). Damaged axons stain darkly with PPD and are not counted. Using this automated method, we found the number of axons counted was within 7% of the number determined manually by two independent observers. To measure the area of the optic nerve, the circumference of the optic nerve was traced in montaged images (40x) of each optic nerve cross-section and the area within this outline was calculated as (mm^2^) using Image J. Axon counts were performed by individuals blinded to the experimental groups.

### Spectral domain optical coherence tomography (SD-OCT)

SD-OCT imaging was performed on eyes with HA + beads and control groups at 4 weeks post-injection using Micron Ⅲ OCT system (Phoenix Research Labs; Pleasanton, CA) under general anesthesia using 1.25% Avertin (2,2,2-tribromoethanol). A series of 50 b-scans were collected and averaged. To see the angle, the OCT probe was aimed vertically at the anterior segment, and the image was centered and focused at the angle.

### Statistical analysis

The effects of ocular hypertension on the HA+beads group, original group, and the HA group were analyzed using ANOVA. A *p < 0*.*05* value was considered statistically significant. All data were presented as mean ± SD.

## Results

### Elevated and sustained IOP in the modified ocular hypertension model

**Fig 1:**
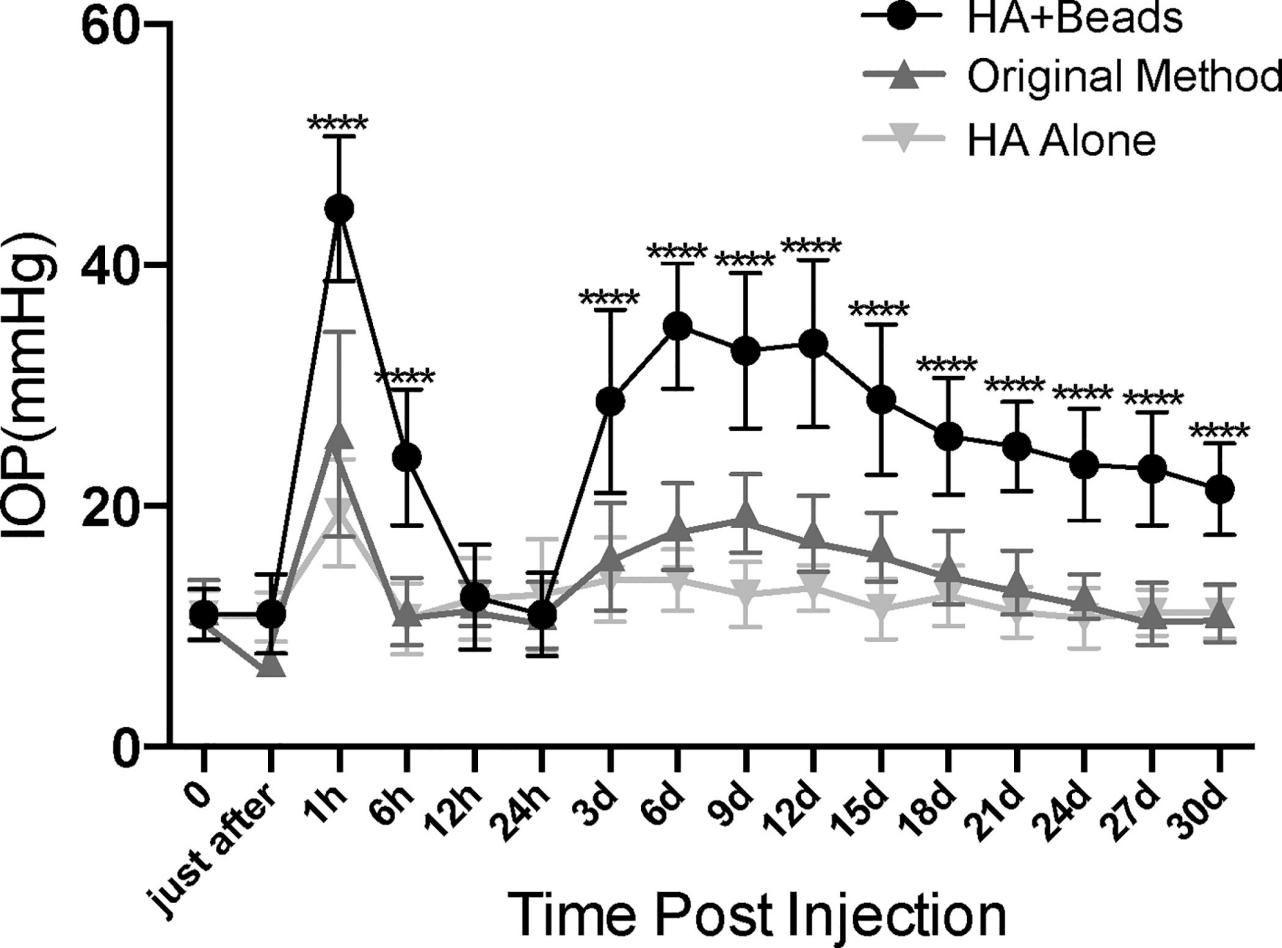
Assessment of IOP elevation after beads injection during the 4 week follow-up period. IOP levels temporarily rose within 20mmHg in the hyaluronic acid (HA) group (HA alone) (n = 13). 3 μl injections of beads suspended in saline (Original Method group) induced a transient elevation of IOP after 1 hour, with a mean peak value of 25.92 ± 8.51 mmHg. When the IOP reached a second peak at 9 days after beads injection, the mean IOP of this second peak did not exceed 20mmHg (n = 13). In the group with 6 μl injections of beads suspended in HA (HA+Beads group), IOP transiently rose to over 40 mmHg at 1 hour post-injection. Although IOP decreased to normal levels 1 day after injection, IOP increased again starting from day 3. IOP reached a second peak between day 6 and day 12. Sustained IOP elevation was observed during the follow-up period (n = 9). Multiple group comparison using ANOVA showed a significant IOP increase at almost all follow-up time points in the mixture (HA+Beads) group relative to the other groups. **** *p* < 0.0001.

To evaluate whether sustained IOP elevation was achieved in hyaluronic acid (HA, Provisc; Alcon Laboratories (S.A.) (Pty) Ltd.) +beads group, compared to original methods group, in which 3 μl of beads in saline was injected, and HA group as a control for HA+beads group, we compared change of IOP among 3 groups. As mentioned earlier, HA was utilized instead of saline for the microbead suspension since HA enabled a slow and sustained release of the microbeads into the anterior chamber and towards the angle over an extended time. To assess post-injection IOP elevation, IOP was measured at the following time points: immediately after, 1 hour, 6 hours, 12 hours, and then every 3 days until 30 days post-injection for each experimental condition. Maximum IOP levels were achieved 1 hours post-surgery in each group (**Fig 1**).

The baseline IOP (before injection), was measured one day prior, and consistent in all groups, with a value of 11.08 ± 2.26 mmHg (n = 35). The mean IOP peak at 1 hours post-injection for each experimental condition are as follows: 44.69 ± 6.00 mmHg in the HA+beads group, 25.92 ± 8.51 mmHg in the original methods group, and 19.44 ± 4.46 mmHg in the HA group. The maximum IOP in the HA+beads group was significantly higher than in the other two groups (*p* < 0.0001). In each experimental group, after reaching the maximum IOP, the IOP returned to baseline levels 24 hours after surgery. The IOP peaked again in both the HA+beads and the original bead method group 6–12 days after injection. The HA+beads group had a second peak value of 34.91 ± 5.21 mmHg, with IOP remaining elevated above 20 mmHg for 4 weeks. At the 30-day post-injection, IOP had gradually decreased to 21.38 ± 3.78 mmHg. In contrast, the original methods group had a second peak value of 19.38 ± 3.26 mmHg, gradually decreasing to a normal IOP level of 11.07 ± 2.42 mmHg at 30 days post-injection. In the HA group, the IOP consistently remained near baseline levels after peaking in the first 1hours post-injection (**Fig 1**).

**Fig 2:**
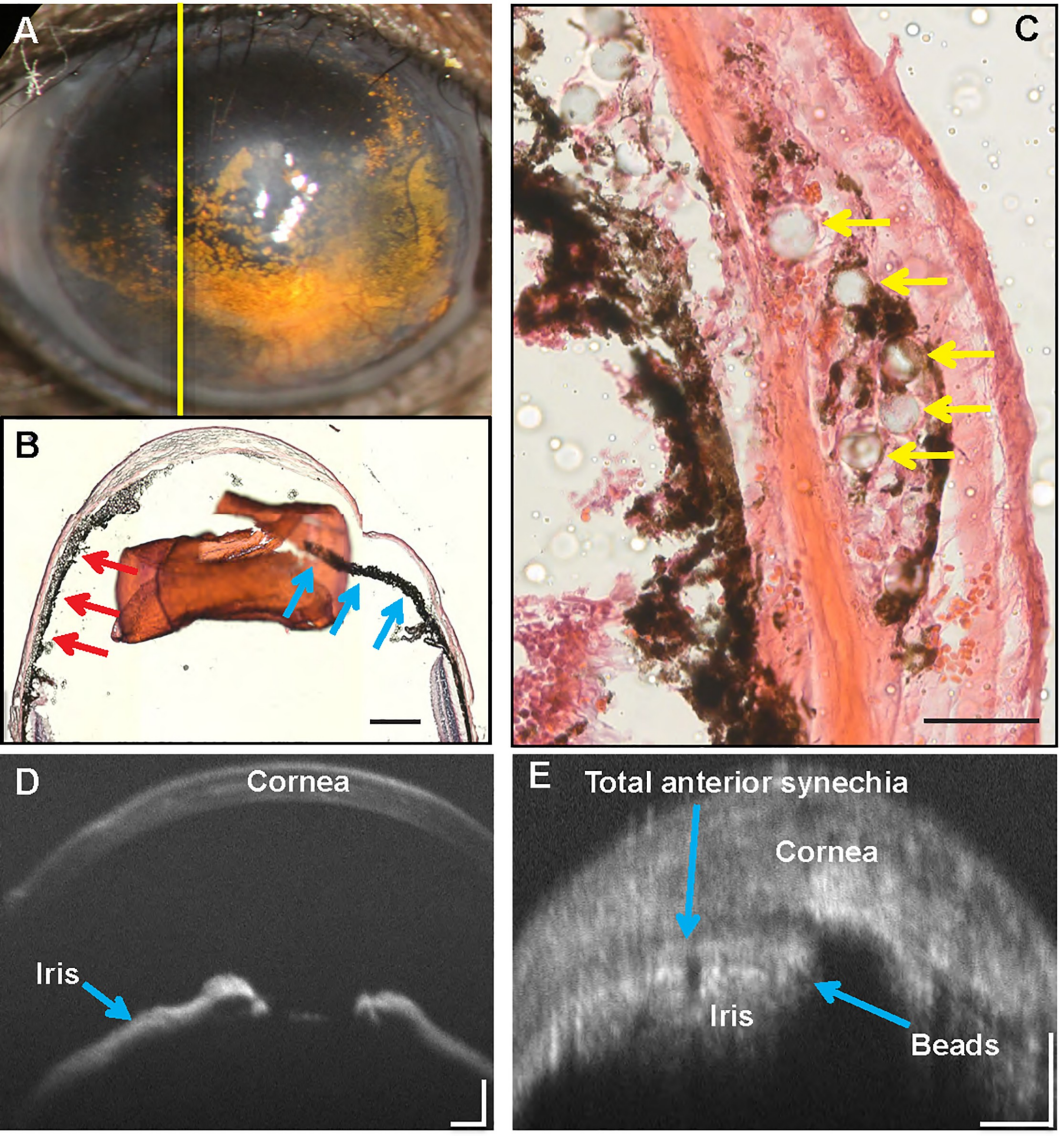
Peripheral synechiae and direct occlusion of Schlemm’s canal by microbeads. **Hematoxylin and Eosin staining of the anterior segment was performed at 30 days post-injection.** (A) Image showed the sliced site of (B), (B) Peripheral synechiae formation with attachment of the corneal endothelium to the inferior iris (red arrows). The superior angle was open (blue arrows). Bar = 500μm. (C) Microbeads (yellow arrows) occluding Schlemm’s canal. Bar = 50μm. (D) Optical coherence tomography (OCT) image of the anterior segment in a normal eye. Bar = 100μm (E) OCT image of the anterior chamber at 30 days post-injection with development of peripheral anterior synechiae. Bar = 100μm.

To determine whether the microbeads occluded the angle, Hematoxylin and Eosin sections of the angle were analyzed. Microbeads led to the development of peripheral anterior synechiae (PAS) 30 days post-injection and the occlusion of Schlemm’s canal, thus blocking the outflow of aqueous humor. Total peripheral synechiae were detected in at least 8 eyes in the HA+beads group (**Fig 2A–2E**).

The optimal suspension volume of microbeads in HA for injection was found to be 6 μl because preliminary experiments using suspension volumes less than 6 μl did not achieve stable elevations of IOP (**S1 Fig**). For example, though a slight elevation of the IOP was observed after an injection of less than 4.0 μl of the suspension, the duration and the maximum value of the elevation were limited. Injection of the suspension with the amount of 4.8 to 5.5 μl led relatively high elevation of IOP, but overall the change of IOP was unstable.

### Enhanced IOP elevation results in increased RGC degeneration

**Fig 3:**
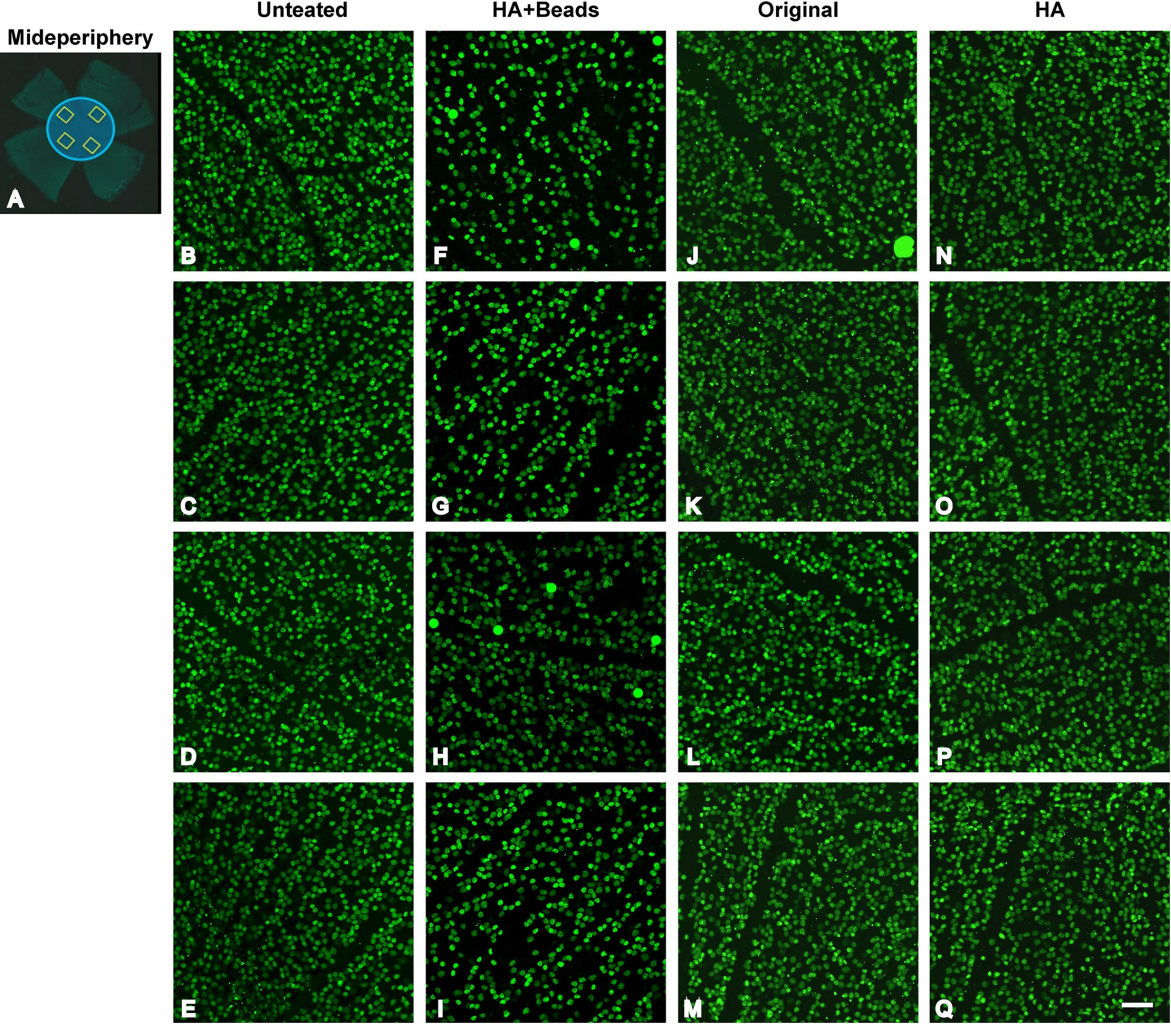
Retinal ganglion cell (RGC) damage in the midperipheral retina from each experimental group. (A) Damage assessment approach. After immunostaining whole retinal flatmounts with Brn-3a, a RGC-specific marker, four areas in the midperiphery (yellow outlined rectangles in the blue circle) were selected to determine RGC density. (B-E) Confocal microscopic images from the selected retinal areas in normal, untreated C57BL/6J mice immunostained with Brn-3a. Confocal images from the hyaluronic acid (HA) + beads group (F-I), the original method group (J-M), and the HA group (N-Q) at 30 days after injection, respectively. Scale bar = 50 μm.

**Fig 4:**
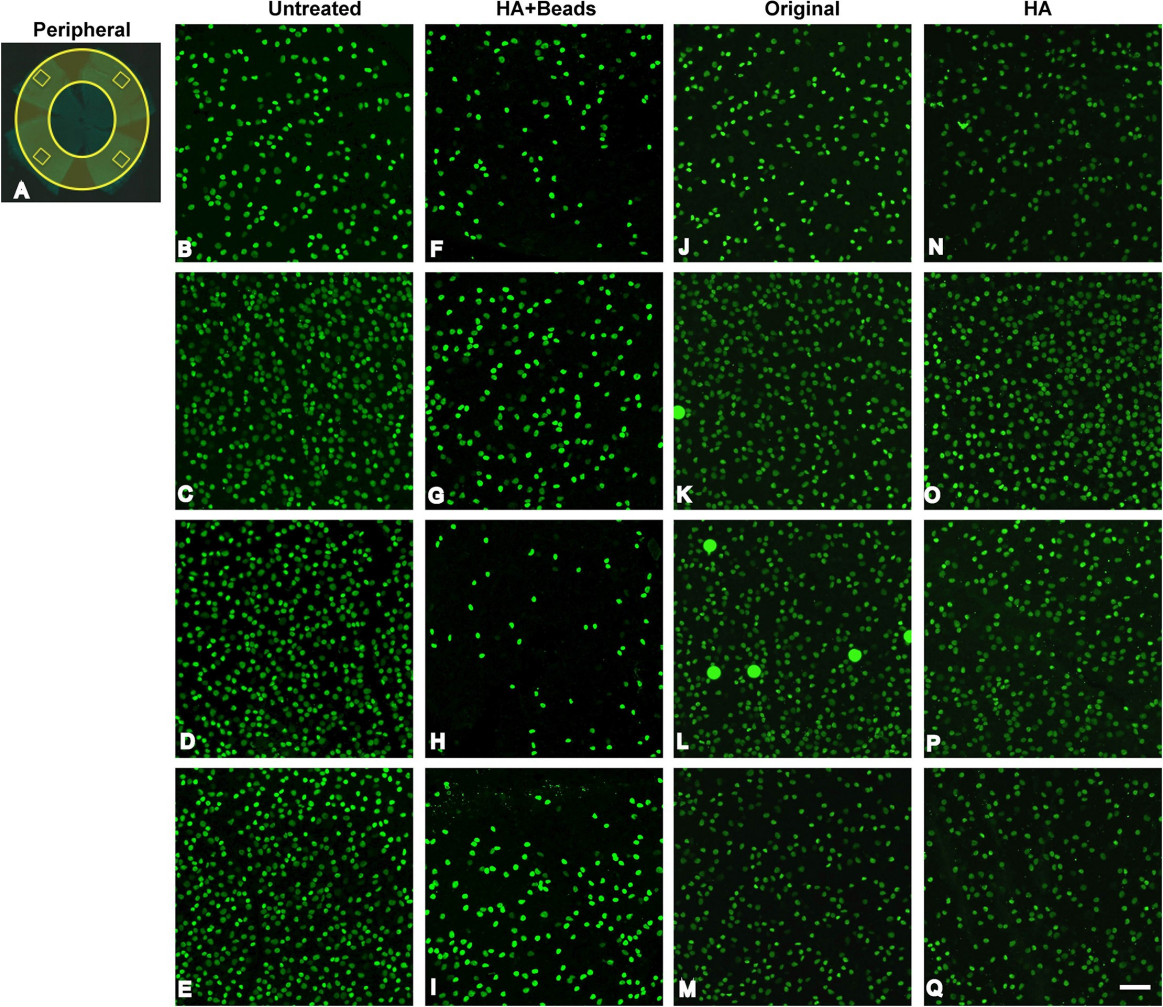
RGC damage in the peripheral retina from each experimental group. **(A) Damage assessment approach.** After immunostaining whole retinal flatmounts with Brn-3a, a RGC-specific marker, four areas in the periphery (yellow outlined rectangles in the yellow circle) were selected to determine RGC density. (B-E) Confocal microscopic images from the selected retinal areas in normal, untreated C57BL/6J mice immunostained with Brn-3a. Retinal selections from the HA+Beads group (F-I), original group (J-M), and HA group (N-Q) at 30 days post-injection. Scale bar = 50 μm.

We compared the number of RGCs among 3 groups as determined by immunostaining of retinas with Brn-3a (**Figs 3 and 4; S3 Fig and S5 Fig**). As described earlier, to separately assess RGC damage in both the midperiphery (**Fig 3A–3Q**) and the periphery (**Fig 4A–4Q**), whole retinas were resected into 4 lobes and stained with Brn-3a. Four areas were selected from each lobe for quantification.

The selected areas were within a circle (blue circle, **Fig 3A**) with a radius of 500 μm around the optic disc. Another four areas were selected in the central area of each peripheral lobe (yellow circle, **Fig 4A**).

**Fig 5:**
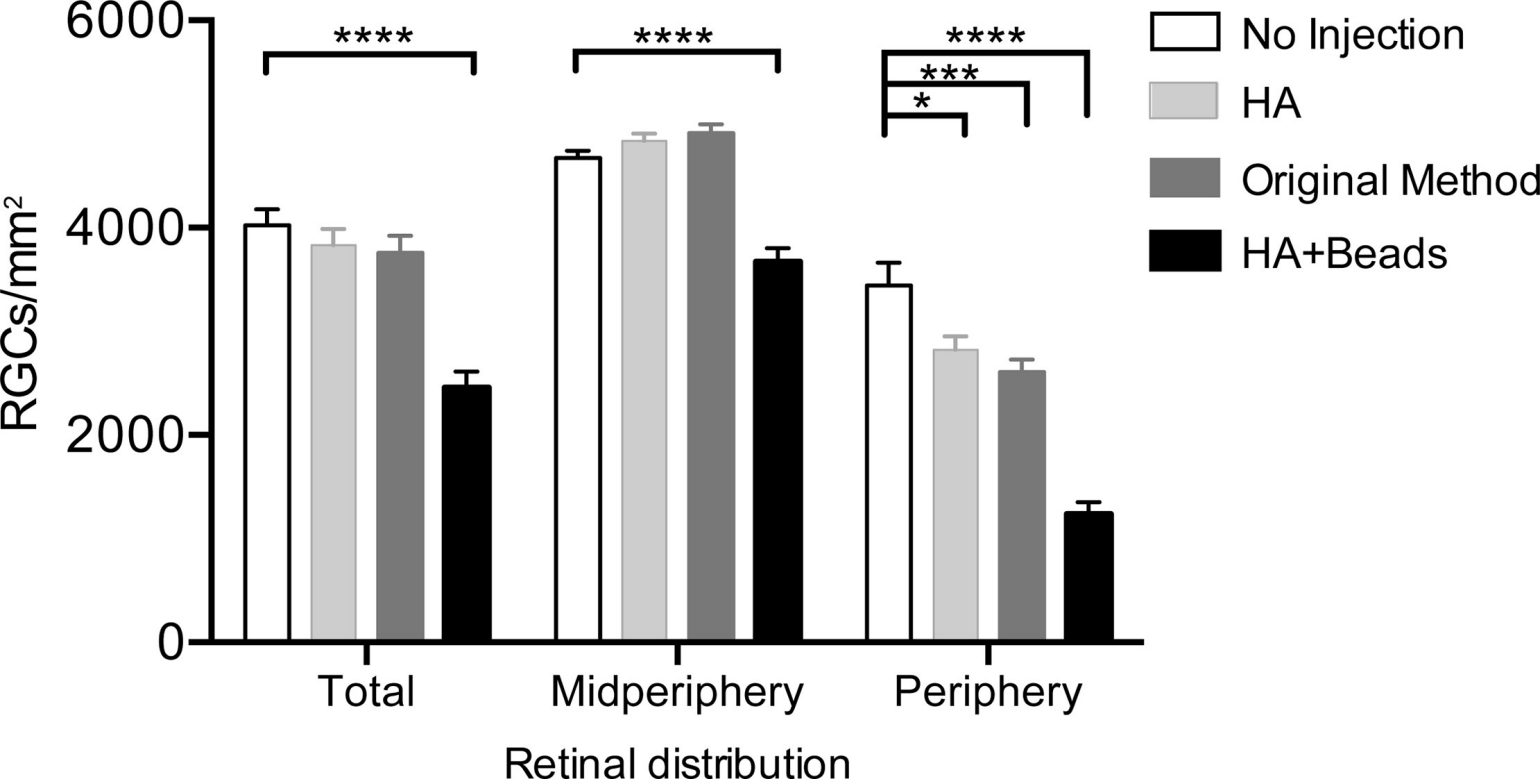
Quantification of RGC damage in different regions of retinal flatmounts immunostained with Brn-3a. Brn-3a staining revealed a significant reduction in peripheral RGC density in each group compared to untreated eyes (white bar). In the hyaluronic acid (HA) + beads group (black bar), significant decreases were also detected in the midperipheral area compared to untreated eyes (*p*< 0.001). Total RGC density was calculated as the average RGC number in both the midperiphery and periphery. **** *p* < 0.0001, *** *p* < 0.001, * *p* < 0.05.

There was a significant reduction (*p* < 0.0001) of 32.4% of midperipheral RGCs in the HA+beads group compared to untreated eyes (*p* < 0.0001) (**Fig 5**).

Maximum damage to RGCs was identified in the periphery in the HA+beads group, with a loss of 64.1% compared to the untreated eyes (*p* < 0.0001) (**Fig 5**). Significant RGC loss in the peripheral retina was found in both the original method and HA groups, with a loss of 24.3% and 18.0% (*p* < 0.001 and *p* < 0.05), respectively. The extent of RGC damage in the original method group of this study was almost the same as in previously reported studies [22, 31] (**Fig 5; Table 1**).

**Table 1:** Survival ratio of RGCs in each experimental group (% relative to the normal untreated group) at 30 days post-injection.

		Total average		Midperiphery		Periphery	
		Survival (%)	P value	Survival (%)	P value	Survival (%)	P value
**normal untreated (n = 4)**		**100**		**100**		**100**	
**HA Alone (n = 7)**		**95.3**	**0.9484**	**103.6**	**0.8799**	**82.0**	**0.0233**
**Original Method (n = 8)**		**93.4**	**0.8196**	**105.1**	**0.6333**	**75.7**	**0.0007**
**HA+Beads** **(n = 11)**	**Total (n = 11)**	**61.1**	**<0.0001**	**78.6**	**<0.0001**	**35.9**	**<0.0001**
	**With CRVO (n = 3)**	**42.2**	**<0.0001**	**63.9**	**0.0013**	**11.2**	**<0.0001**
	**Without CRVO (n = 8)**	**68.1**	**<0.0001**	**84.2**	**<0.0001**	**50.0**	**<0.0001**

RGC damage in the Hyaluronic acid (HA) + Beads group was further categorized into two groups: with or without central retinal vein occlusion (CRVO). Within the HA+Beads group, eyes without CRVO were found to have a mean RGC loss (defined as 1 –survival ratio) of 31.9% (range of 25.8–80%) at 30 days post-injection.

RGCs stained with Brn-3a revealed that the distribution of RGC damage spread to at least two lobes of the peripheral retina in the HA+beads group (**S4 Fig and S5 Fig**).

### Suspected central retinal vein occlusion

**Fig 6:**
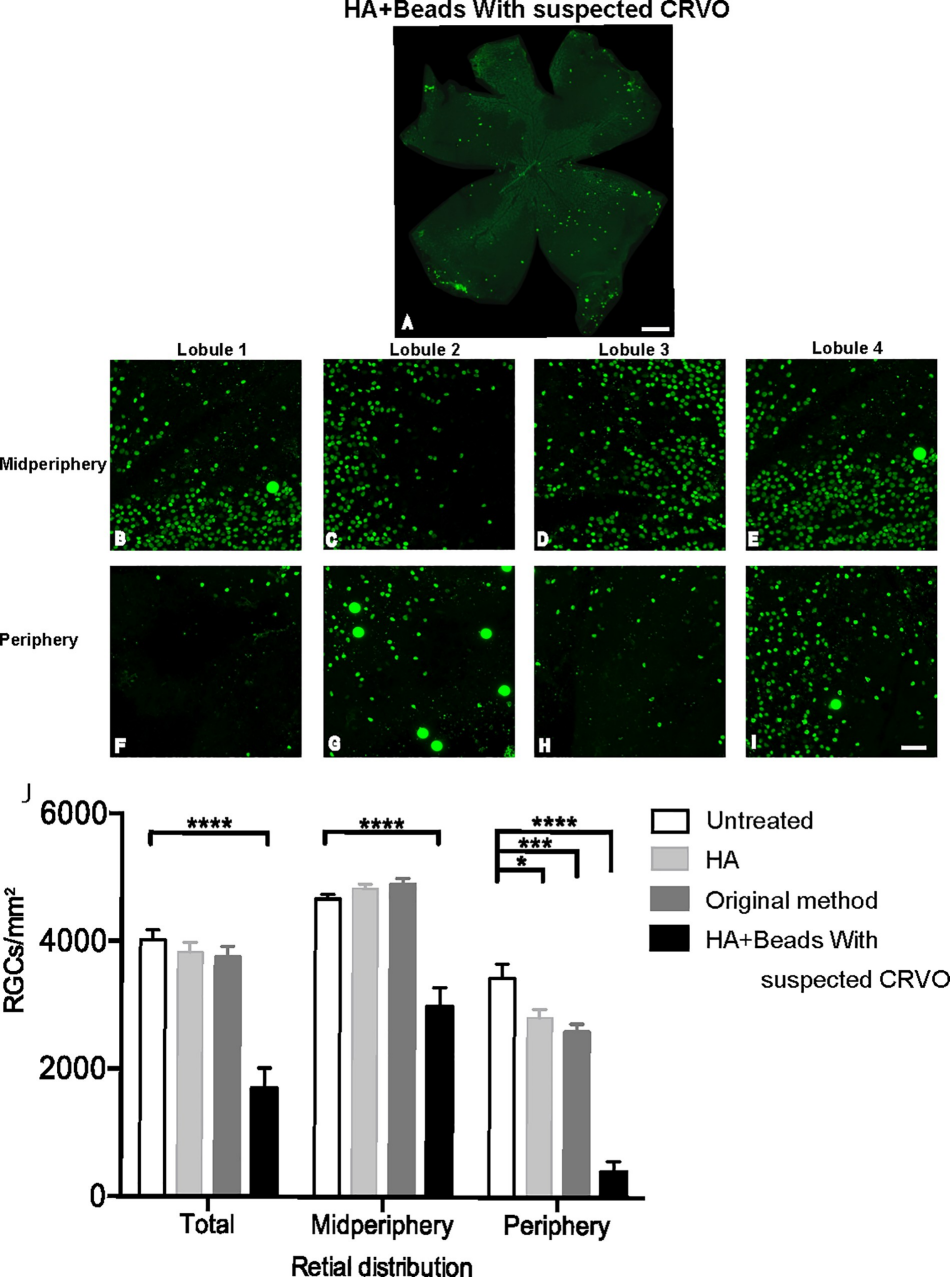
Visualization of RGCs in Brn-3a immunostained retinas from the hyaluronic acid (HA) + beads group with suspected central retinal vein occlusion (CRVO). (A) Extensive RGC damage in all peripheral lobes and blotched loss of RGCs in the midperipheral retina were observed. Scale bar = 500 μm. (B-E) Confocal microscopic images of retinal selections in the midperiphery of each lobe at 30 days post-injection. (F-I) Retinal selections in the periphery of each lobe. Scale bar = 50 μm. (J) Quantification of RGC damage of retinal flatmounts immunostained with Brn-3a from the HA+Beads group with suspected CRVO (n = 3). The eyes with suspected CRVO in the HA+Beads group showed very severe RGC damage in both the periphery and midperiphery at 30 days post-injection. **** *p* < 0.0001, *** *p* < 0.001, * *p* < 0.05.

In 3 out of the 11 eyes (27%) in the HA+beads group, a complication of the model manifested in the form of potential central retinal vein occlusion (CRVO) (**Fig 6; S1 Table**).

**Fig 7:**
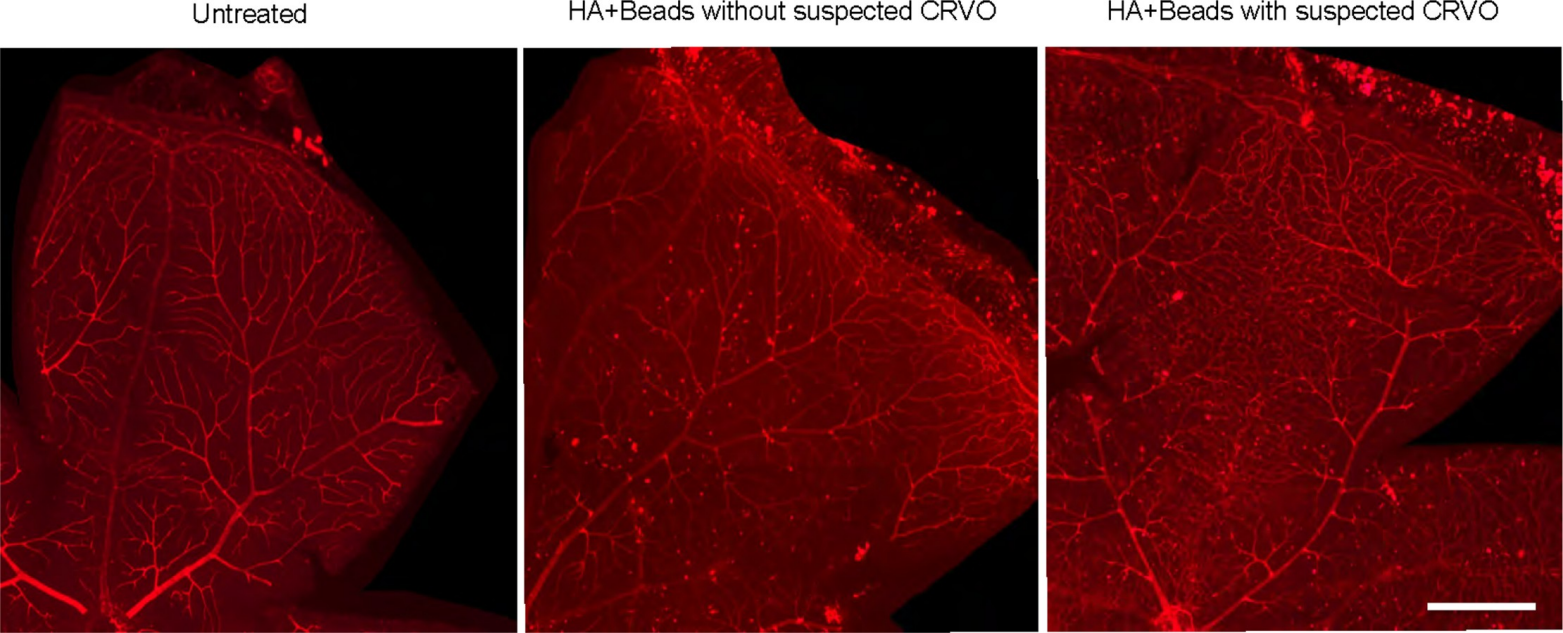
**Lectin-stained retinas of the (A) normal, untreated group, (B) hyaluronic acid (HA)+Beads group without suspected CRVO, and (C) HA+Beads group with suspected CRVO.** (B) Slightly dilated and tortuous vessels were observed in the distal area 30 days post-injection. (C) Tortuous vessels and small dilated vessels among large to middle-sized vessels were observed from the optic disc to the peripheral retina. Scale bar = 500 μm.

In these cases, lectin staining revealed the development of tortuous and dilated retinal vessels throughout the whole retina after inducing OH. Additionally, RGC damage was markedly severe in the midperiphery as well as in the periphery. These changes were highly suggestive of CRVO, because the tortuous and dilated patterning of retinal vessels throughout the entire retina after inducing OH was similar to the vascular patterning seen with fluorescein angiography in cases of CRVO [32] [33] [34]. In contrast, no dilation or tortuous vessels were observed in both untreated eyes and in the other 8 out of 11 eyes in the HA+beads group (**Fig 7**).

The presence of suspected CRVO was correlated with severe RGC damage, with Brn-3a staining revealing significant loss of RGCs in both the midperipheral and peripheral retina (**Fig 6B–6I**). A total loss of 88.8% of RGCs was identified in the periphery of eyes with suspected CRVO, while a loss of 36.1% was identified in the midperiphery (**Fig 6J; Table 1**). Of note, even when the samples with suspected CRVO were excluded from analysis, a significant reduction in RGCs was observed in both the peripheral and midperipheral retina, with losses of 50.0% and 15.8%, respectively (*p* < 0.0001 in both areas). Even HA+beads without suspected CRVO was able to induce more severe damage in RGCs in both midperiphery and periphery, compared to the damage in the original method group, with losses of 24.3% and 0%, respectively (**S6 Fig, Table 1**).

### Axon loss

**Fig 8:**
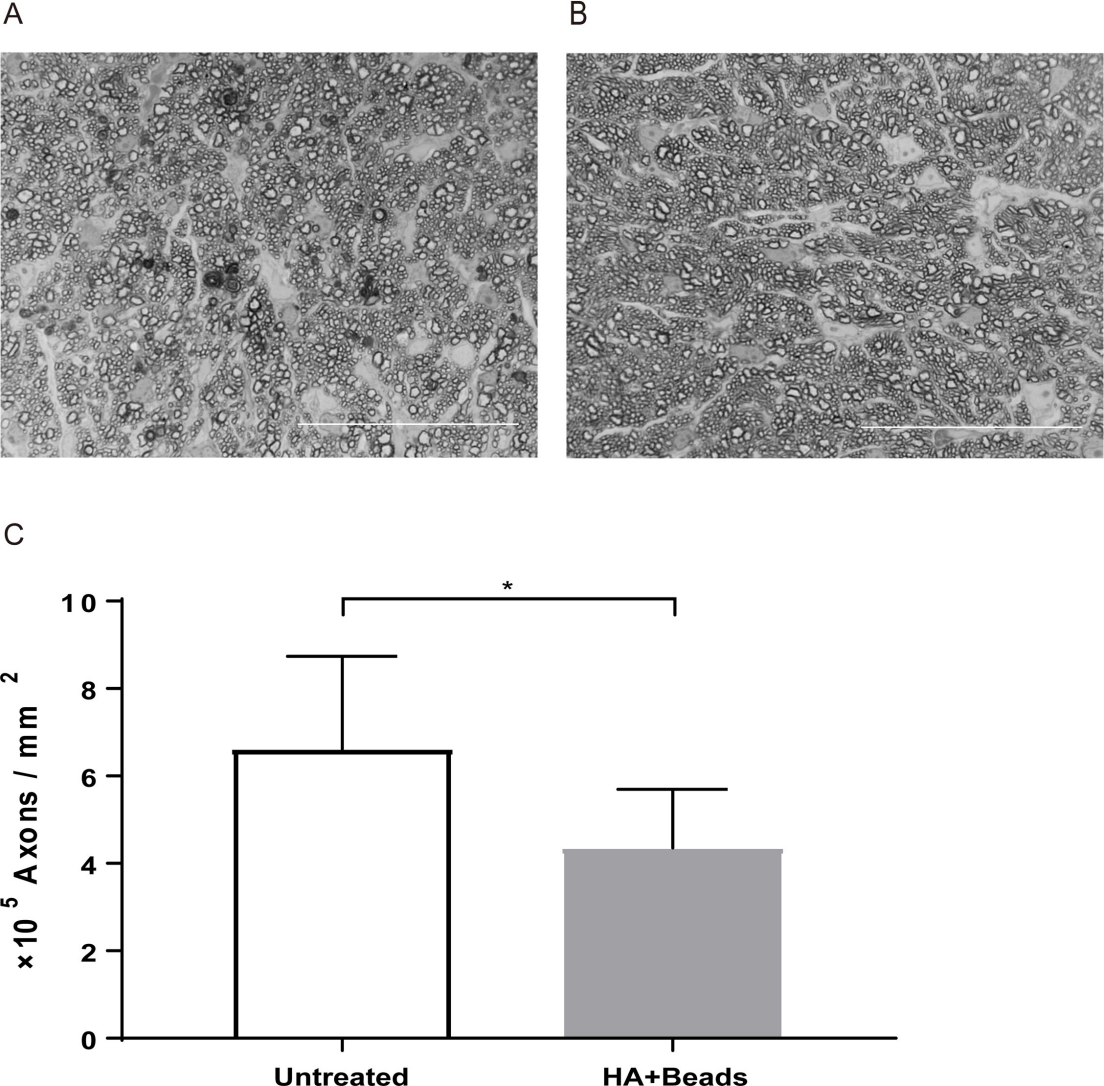
Axon loss of at 30 days post inducing ocular hypertension. (A) The axon damage of hyaluronic acid with beads injected eye (HA with beads group). (B) The axon in untreated eye. (C) Comparison of axonal damage between hyaluronic acid (HA) + beads group (n = 5) and untreated group (n = 4). *p < 0.001. Scale bar = 50μm.

We analysed survival axonal density in optic nerve and we compared it between HA+beads and untreated group at 30 days post inducing ocular hypertension. The mean axonal density in HA+beads group was 4.35 ± 1.35 × 10^5^ / mm^2^ and that in untreated group was 6.62 ± 2.12 × 10^5^ / mm^2^. There was a statistically significant difference in the mean axon density between HA+beads and control group (*p* < 0.01). (**Fig 8**)

## Discussion

A long-lasting and reproducible elevation of IOP was successfully obtained by injection of microbeads suspended in hyaluronic acid, followed by closure of the incision site with WEBGLUE. This complete patch at the incision site likely contributes to the initial IOP spike observed during the first 24 hours after injection and a relative induction of hypoxia. In turn, this first IOP peak may account for the significantly greater loss of RGCs after 4 weeks than that observed in other previously described microbead occlusion models. RGC damage in the peripheral retina was more severe compared to that in the midperipheral retina. These observations suggest that RGCs in the peripheral retina are more sensitive to IOP elevation than those in the midperiphery, or that RGCs in the midperiphery have a higher tolerance for IOP elevation than those in the periphery.

Currently, elevation of IOP induced by microbead occlusion in mice has been explored in several studies [21, 27, 28]. However, these models do not lead to sustained elevation of IOP with values over 20 mmHg. Moreover, in the original bead-only model, damage to RGCs is fairly limited during in the 4 to 6 week follow up period (**S7 Fig**). In general, there is considerable variability in the concentration of beads remaining in the eye immediately following injection in these previously reported models. This variability in microbead concentration after injection may be primarily due to leakage of the injected beads, which were suspended in saline or PBS, from the incision site. Leakage is prevented in our model due to the use of HA and WEBGLUE, as a result, prior models may have been difficult to reproduce accurately and consistently due to bead loss and outflow; respectively, enhancing the bead-induced blockage and reducing normalization of the IOP immediately post-injection.

Several researchers have attempted to inject beads suspended in viscoelastic substances, such as hyaluronic acid, into the anterior chamber. Quigley et al. have explored injections of 2–4μl of 1–6μm beads and 1–3 μl of HA, which resulted in a maximum IOP of about 40 mmHg at 7 days post-injection. The timing and magnitude of this peak IOP value is similar to our results, however RGC loss was limited in their study. They did not use glue at the incision site, and it could be one of the reasons for no apparent acute IOP spike within hours after the injection. [22, 35–37] Smedowski et al. proposed a model in which 15 μl of beads with viscoelastic material induced elevated IOP in rats [38]. The microbead mixture contained two kinds of particles, with diameters of 6.0 μm and 10 μm, respectively. Although this model showed sustained IOP, RGC loss was only about 33% compared to untreated eyes, and the damaged areas were not specified. Matsumoto et al. injected microbeads with sodium sulfate-sodium hyaluronate via a scleral incision site using a 34-gauge needle in rats [39]. The IOP changes measured by this group after 1 week were quite similar to our results. Of note, this group did not use glue at the incision site, and no IOP spike was observed during the first 24 hours. In fact, the anterior segment at 4 weeks post surgery differed in appearance from our findings (**See S8D Fig**). These differences suggest that the initial IOP peak during the first 24 hours obtained with our methodology may play an important role in inducing significant RGC damage. Thus, we believe that it is important to seal the incision site with glue immediately after the microbead mixture is injected in order to prevent backflow of beads. Furthermore, in order to induce greater damage to RGCs, we adjusted the concentration of beads in hyaluronic acid before injection (**See S2 Fig**). Strengths and weaknesses of the original microbead occlusion method as well as our method are listed in **Table 2**.

**Table 2:** Pros and Cons between the original and modified method.

Type	original method [21, 40, 41]	modified method
Pros	Clarity of anterior chamber	Sustained elevation of IOP Easy and consistent reproducibility Increased damage to RGCs: 64.1% loss in peripheral retina
Cons	IOP relatively low Inconsistent reproducibility Minimal damage to RGCs: 24–43% loss	Bleeding (10 days post-injection) Opacity of cornea and anterior chamber Suspected CRVO

Recently Ito et al. injected magnetic microbeads in mice [42] and the group successfully sustained high IOP with the value of about 20 mmHg during 6 weeks.

RGC damage in this study was relatively severe, with a loss of 64.1% at the periphery and 21.4% at the midperiphery, as compared to previous studies [22, 27, 28, 31, 38, 43]. RGC loss averaged over the whole retina was 38.9% at 4 weeks post-injection. But as was described earlier, this damage assessment included 3 out of 11 eyes that developed suspected CRVO after injection with 6.0 μl of the bead mixture. The damage in eyes with suspected CRVO (n = 3) was removed from analysis, and there was still a significant reduction in RGCs in both the peripheral and midperipheral retina, with losses of 50.0% and 15.8% respectively (*p*< 0.0001 in both areas). RGC damage averaged over the entire retina was 31.9% (**S6 Fig; Table 1**).

A potential limitation of this method is that the injection of 6.0 μl of beads with HA induced bleeding in the anterior chamber 6 to 14 days after injection. Although suspected CRVO did not develop in all eyes, bleeding occurred around 7 to 14 days post-injection in all eyes. It has been reported that inflammation occasionally accompanies laser-induced elevation of IOP in mice [20]. Additionally, lectin staining showed that the injection of 6.0 μl of beads with HA induced the tortuosity and dilation of retinal vessels. Based on these findings, the observed bleeding may be a consequence of inflammation and partial ischemia induced by sustained increases in IOP, which may also account for the neovascularization that was identified in the deep cornea during the follow-up period in the HA+beads group (**S8 Fig**).

It is important to recognize that clinically acute angle-closure glaucoma (AACG) is sometimes accompanied by CRVO [44–47], which induces severe visual impairment [44]. It is possible that the first spike of IOP (around 50 mmHg) with our methodology may have evoked a disturbance in the retinal circulation that led to RGC damage, even though complete ischemia from the retinal circulation has been shown to occur when the IOP is raised over 70 mmHg [48–50]. Rovere described the responses of RGCs and melanopsions in the acute OH model [51]. In this study, RGC damage in the periphery of the retina was more severe than that in the midperiphery (**Figs 3–5; S4 Fig and S5 Fig; Table 1**) showed a decreased distribution of RGCs in the peripheral area using a laser-induced ocular hypertension model [52]. Clinically, it has been suggested that peripheral damage in the visual field is correlated with glaucomatous damage, which was accompanied with the damage of retinal nerve fiber layer or optic disc glaucomatous changes [53–56]. Moreover, eyes with chronic angle-closure glaucoma suffer from more damage in the nasal area of the visual field compared to eyes with normal tension glaucoma, which worsens the visual field in the central area [57]. Taken together, these findings support the idea that elevated IOP may damage the RGCs of the peripheral retina.

A limitation is the variability of IOP around 3 to 14 days post-injection. Corneal damage was usually induced by the elevation of IOP during this period. Although a previous report showed IOP measurement using the TonoLab tonometer does not correlate with corneal thickness [58], pathological corneal thickness may have an effect on measurement of IOP with the TonoLab tonometer. Another limitation of this model is that ERG and retinal OCT, critical tools for glaucoma studies, could not be applied to this model due to the opacity of the anterior chamber and corne.

## Conclusions

In summary, we developed a glaucoma model which improved on the existing microbead injection model by mixing beads with hyaluronic acid, this induced sustained elevation of IOP and enhanced RGC damage with greatly improved replicability. The pattern of RGC damage in this model provides a better understanding of RGC death in the setting of ocular hypertension and the clinical course of acute angle-closure glaucoma.

## Supporting information

S1 Fig Comparison of IOP elevation with different injection volumes of beads suspended in hyaluronic acid (HA) during the 4 week follow-up period. Injection volumes of 3.5, 4.0, 4.8, 5.0, 5.5, and 6.0 μl of beads mixed with HA were assessed. Additionally, IOP was measured in the conventional method (original) group, which uses 3.0 μl of beads in saline. Injection volumes from 3.5 to 5.5 μl did not lead to sustained IOP elevation while the 6 μl group had the highest sustained IOP profile.
(PDF)

S2 Fig Sequential photographs of the entire injection procedure, from making the incision site to closing the site with glue. (A) Cutting of the upper eyelashes. (B) Making the superotemporal incision site using a 31G needle. (C-E) 6.0μl of microbeads suspended in hyaluronic acid was gradually injected into the anterior chamber using glass micropipettes. (F) Incision site was patched with glue.
(PDF)

S3 Fig Brn3a stain of the whole retina in a normal, untreated eye. To analyze RGC damage, we divided the area into the midperiphery (Blue circle) and periphery (circular area outlined in yellow) because RGC density in the midperiphery (A) and periphery (B) are quite different. 4 areas in each region (red squares) were selected and RGC damage was estimated by the automated cell counting function in the ImageJ software.
(PDF)

S4 Fig Heat map visualization of RGC density in each group 30 days post-injection. Red corresponds to over 3275 RGCs/mm2 present in the region. Green corresponds to 1640 RGCs/mm2 RGCs. (A) Typical RGC damage in the hyaluronic acid (HA) + beads group without suspected CRVO. RGC damage was induced in at least 2 lobes of the peripheral retina. (B) Typical RGC damage in the HA+Beads group with suspected CRVO. RGC damage severely happened in whole retina. (C) Moderate RGC damage was observed in 1 to 2 lobes of the peripheral retina in the HA group. (D) Mild RGC damage was induced in 1 to 2 lobes of the peripheral retina in the original method group.
(PDF)

S5 Fig Typical RGC damage seen in whole retinas immunostained with Brn-3a, a specific marker of RGCs, from the Hyaluronic acid (HA) + beads group. Peripheral retinal damage in 2 of the lobes was prominent. Bar = 500μm.
(PDF)

S6 Fig Quantification of RGC damage of retinal flatmounts immunostained with Brn-3a from the hyaluronic acid (HA) + beads group without suspected CRVO (n = 8). **** *p* < 0.0001, *** *p* < 0.001, * *p* < 0.05.
(PDF)

S7 Fig Original microbead occlusion model in C57BL/6J (B6) mice. (A) IOP change in B6 mice after injection with 3μl of beads suspended in saline as compared to saline-injected eyes (without beads). Maximum IOP was observed around 7–10 days, with a mean value of 26.83 ± 3.84 mmHg in the B6 mice with beads group. (B) Quantification of RGC density using Tuj-1 stain in untreated (Untreated) and beads-injected (Original Method) B6 mice at 30 days post-treatment. There was a significant reduction in the number of RGCs in the original method group compared to the normal,untreated eye group. Nevertheless, RGC damage was overall minimal. (C) RGC immunostaining with Tuj1 from a normal, untreated B6 mouse. (D) RGC immunostaining with Tuj1 from a beads-injected B6 mouse at 30 days post-injection. Scale bar = 50 μm.
(PDF)

S8 Fig Potential limitations of anterior images immediately after injection and 1 month post-injection. (A-C) Anterior segment photographs just after injection in each experimental group. (D) Neovascularization was identified in the deep cornea in the hyaluronic acid (HA) + Beads group. (E) Beads were identified inferonasally in the original method group. (F) Anterior segment photographs in the HA group were essentially normal.
(PDF)

S1 Table Prevalence of suspected central retinal vein occlusion (CRVO) in each experimental group. In the hyaluronic acid (HA) + Beads group, suspected central retinal vein occlusion (CRVO) occurred in 27% of eyes (3/11 eyes), even though suspected CRVO was not observed after induction of ocular hypertension (OH) in the other experimental groups.
(DOCX)

## Abbreviations

RGC: retinal ganglion cell
IOP: intraocular pressure
PAS: peripheral anterior synechiae
HA: hyaluronic acid
CRVO: central retinal vein occlusion
AACG: acute angle-closure glaucoma
OCT: optimal cutting temperature
HE: Hematoxylin and Eosin
PFA: paraformaldehyde
PPD: Paraphenylene diamine
