# Supplementary figures and images for: Mouse model of ocular hypertension with retinal ganglion cell degeneration
Source: PLoS One. 2019 Jan 14;14(1):e0208713. doi: 10.1371/journal.pone.0208713 (PMC6331128; doi:10.1371/journal.pone.0208713)

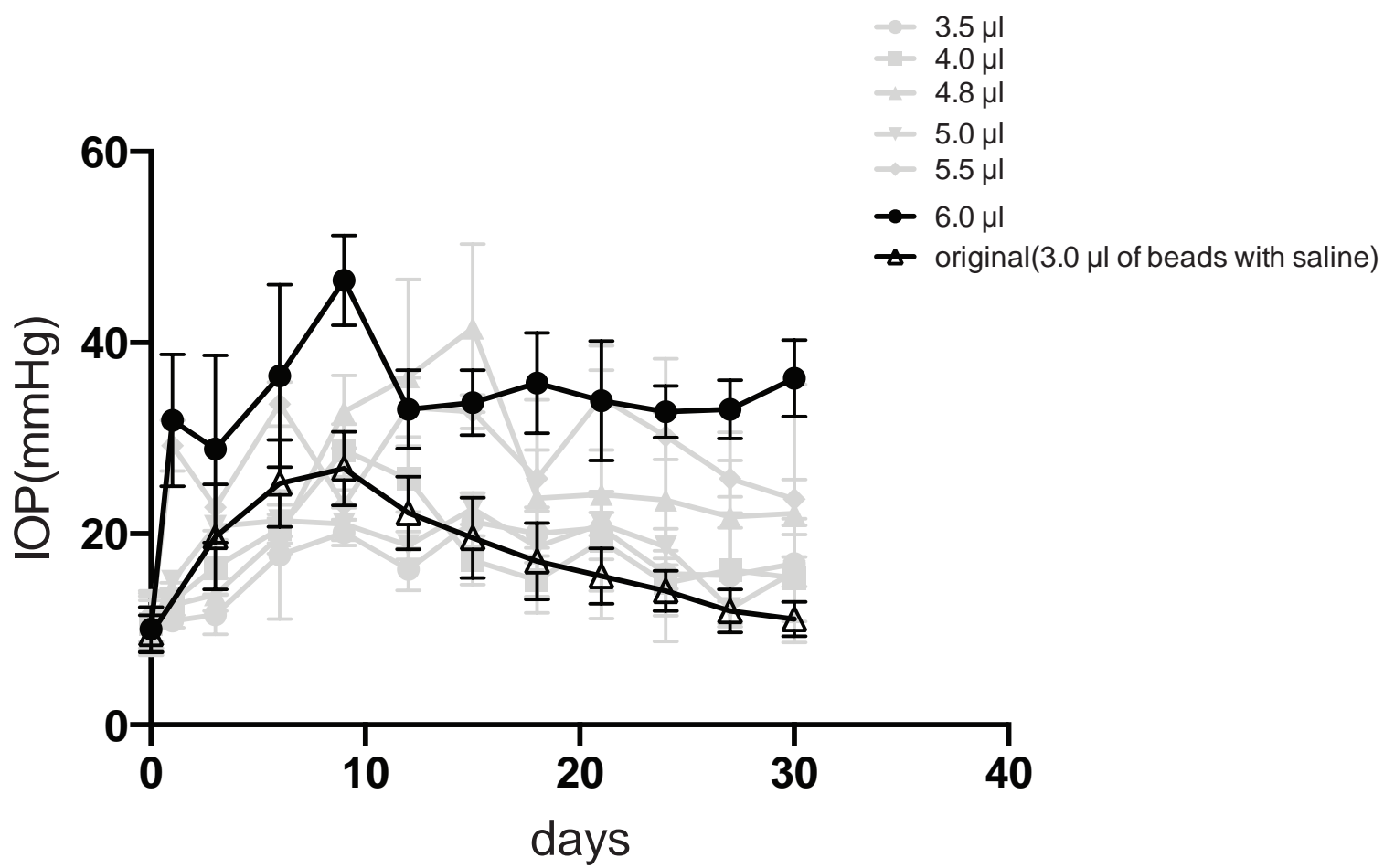

Supplement: S1 Fig — Injection volumes of 3.5, 4.0, 4.8, 5.0, 5.5, and 6.0 μl of beads mixed with HA were assessed. Additionally, IOP was measured in the conventional method (original) group, which uses 3.0 μl of beads in saline. Injection volumes from 3.5 to 5.5 μl did not lead to sustained IOP elevation while the 6 μl group had the highest sustained IOP profile. (PDF) [file pone.0208713.s001.pdf]

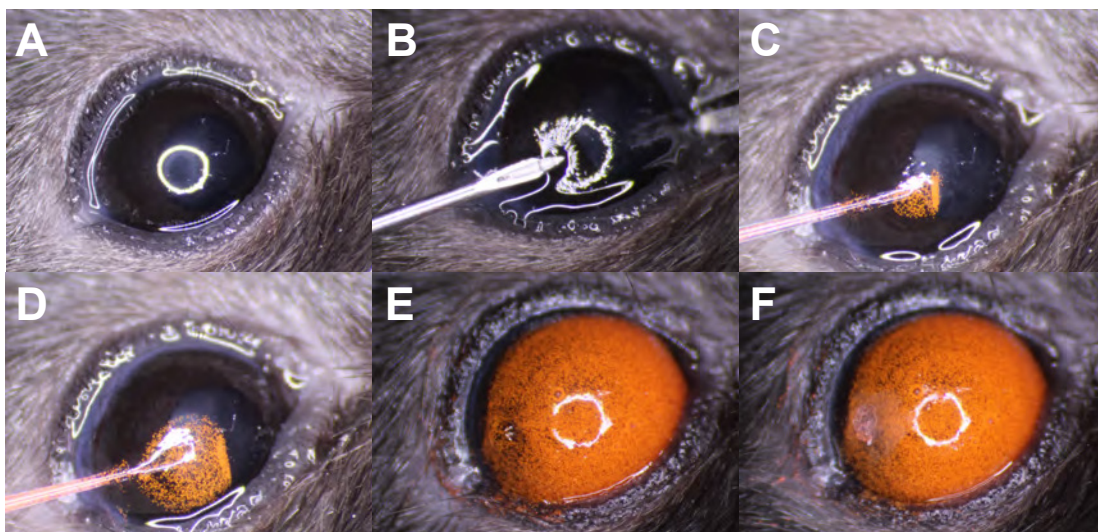

S2 Fig

Supplement: S2 Fig — (A) Cutting of the upper eyelashes. (B) Making the superotemporal incision site using a 31G needle. (C-E) 6.0μl of microbeads suspended in hyaluronic acid was gradually injected into the anterior chamber using glass micropipettes. (F) Incision site was patched with glue. (PDF) [file pone.0208713.s002.pdf]

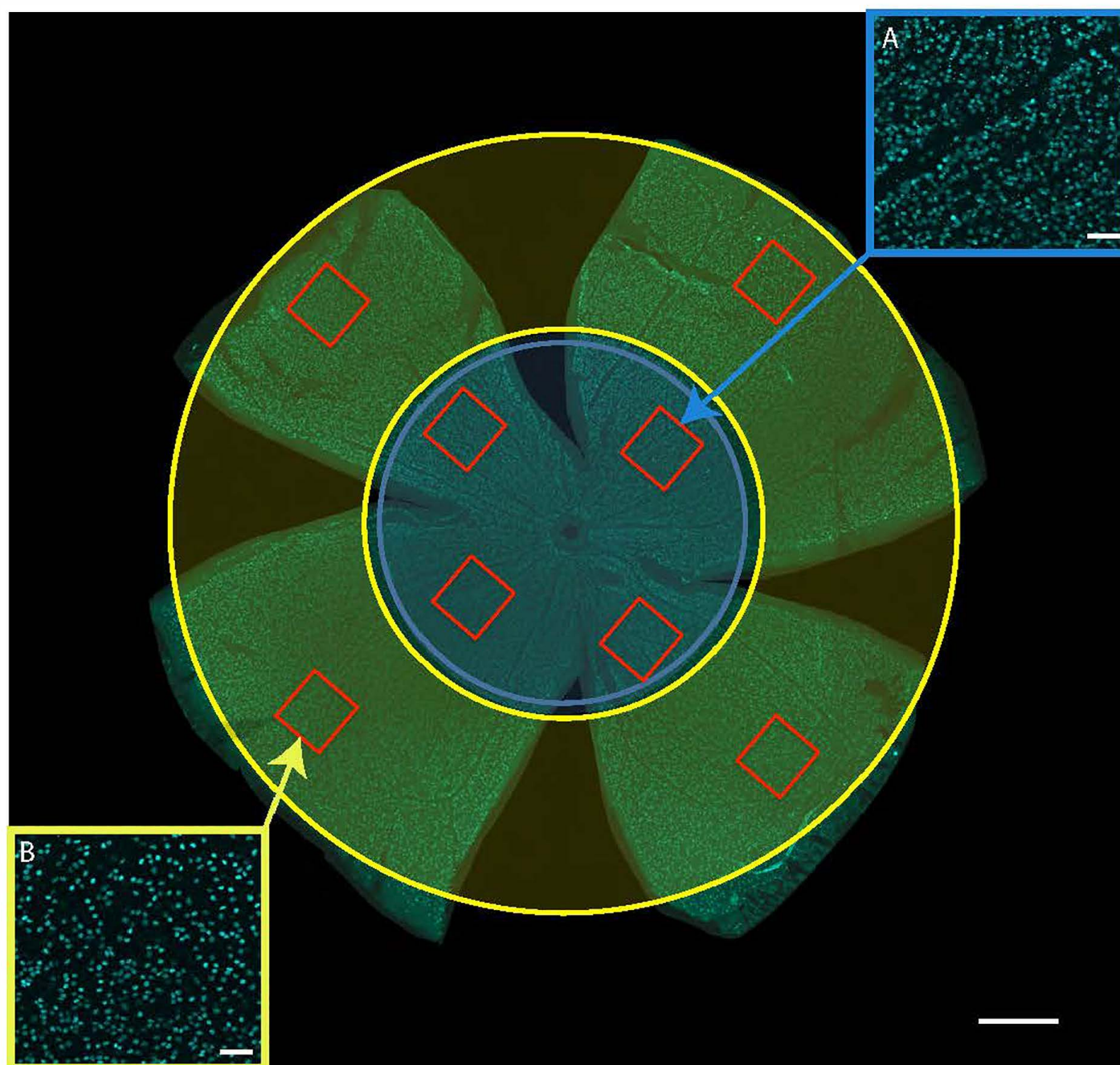

S3 Fig

Supplement: S3 Fig — To analyze RGC damage, we divided the area into the midperiphery (Blue circle) and periphery (circular area outlined in yellow) because RGC density in the midperiphery (A) and periphery (B) are quite different. 4 areas in each region (red squares) were selected and RGC damage was estimated by the automated cell counting function in the ImageJ software. (PDF) [file pone.0208713.s003.pdf]

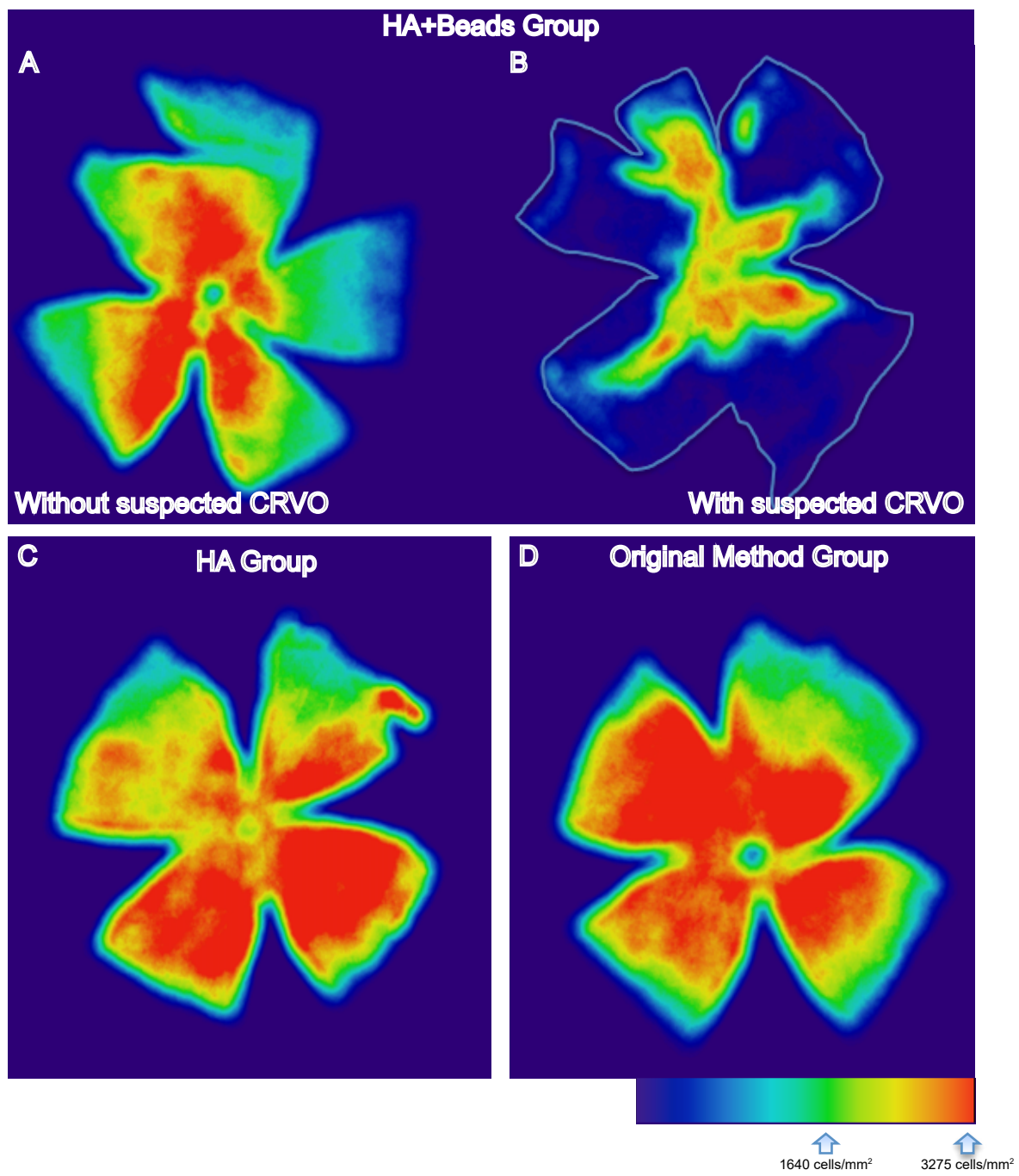

S4 Fig

Supplement: S4 Fig — Red corresponds to over 3275 RGCs/mm2 present in the region. Green corresponds to 1640 RGCs/mm2 RGCs. (A) Typical RGC damage in the hyaluronic acid (HA) + beads group without suspected CRVO. RGC damage was induced in at least 2 lobes of the peripheral retina. (B) Typical RGC damage in the HA+Beads group with suspected CRVO. RGC damage severely happened in whole retina. (C) Moderate RGC damage was observed in 1 to 2 lobes of the peripheral retina in the HA group. (D) Mild RGC damage was induced in 1 to 2 lobes of the peripheral retina in the original method group. (PDF) [file pone.0208713.s004.pdf]

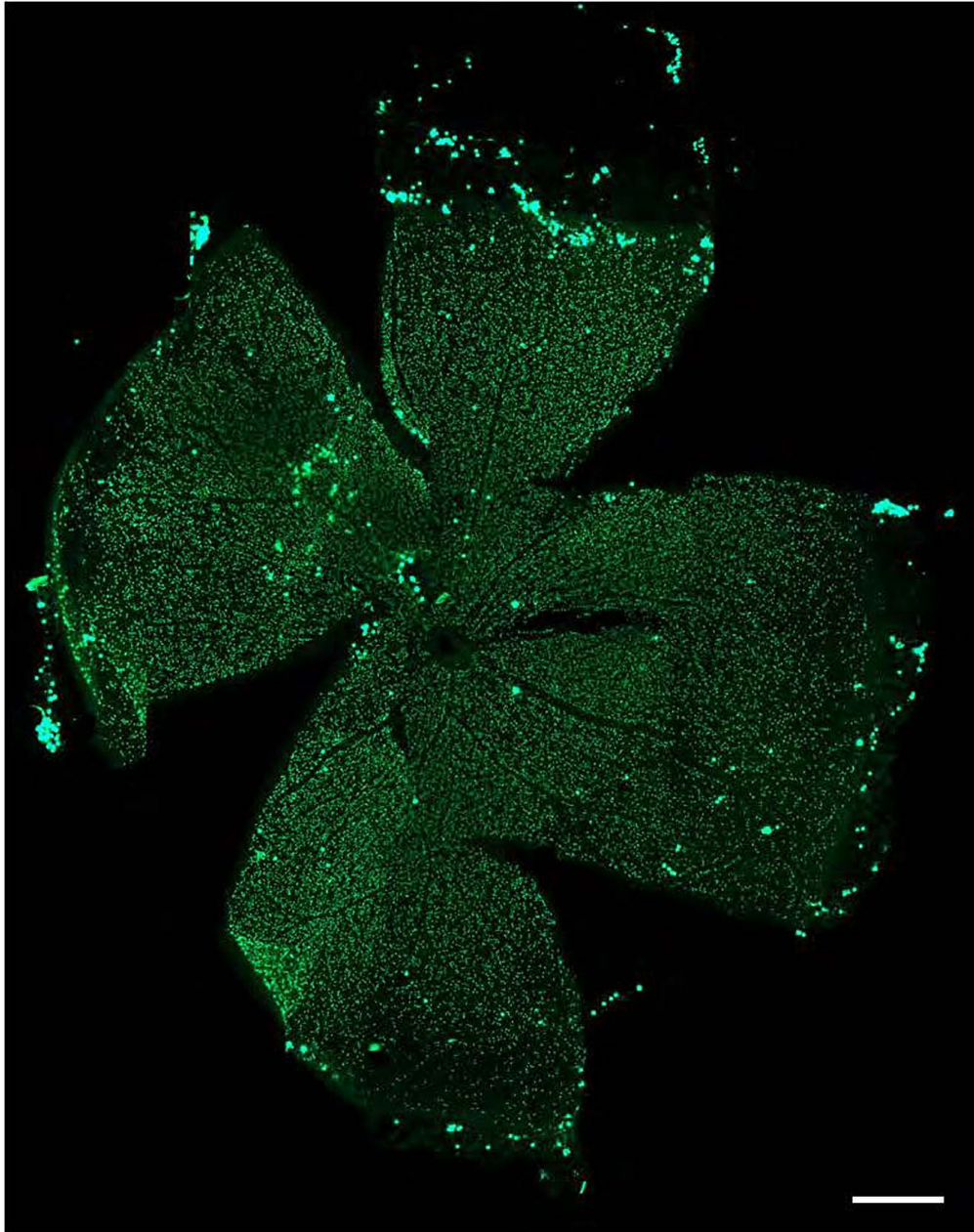

Supplement: S5 Fig — Peripheral retinal damage in 2 of the lobes was prominent. Bar = 500μm. (PDF) [file pone.0208713.s005.pdf]

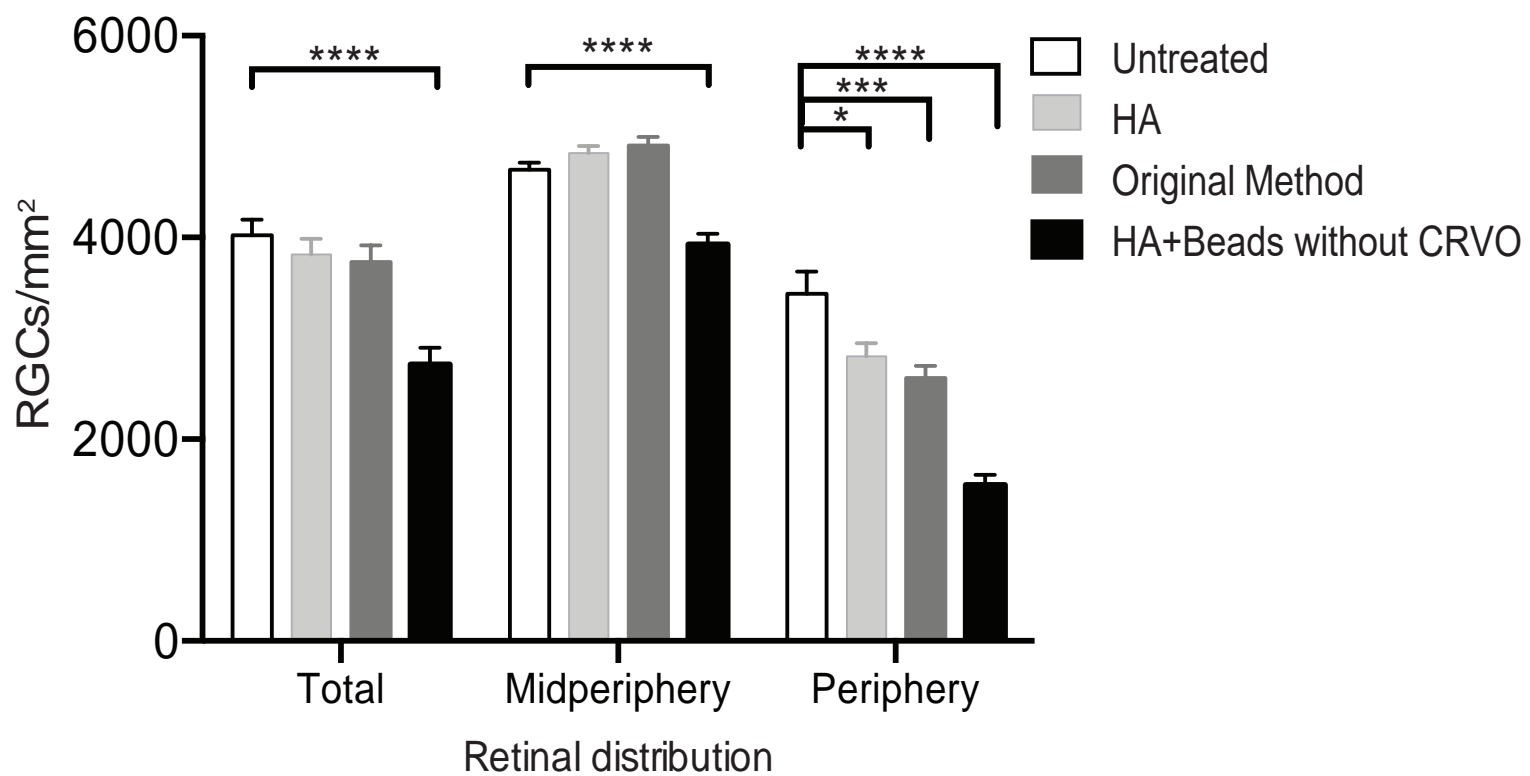

Supplement: S6 Fig — **** p < 0.0001, *** p < 0.001, * p < 0.05. (PDF) [file pone.0208713.s006.pdf]

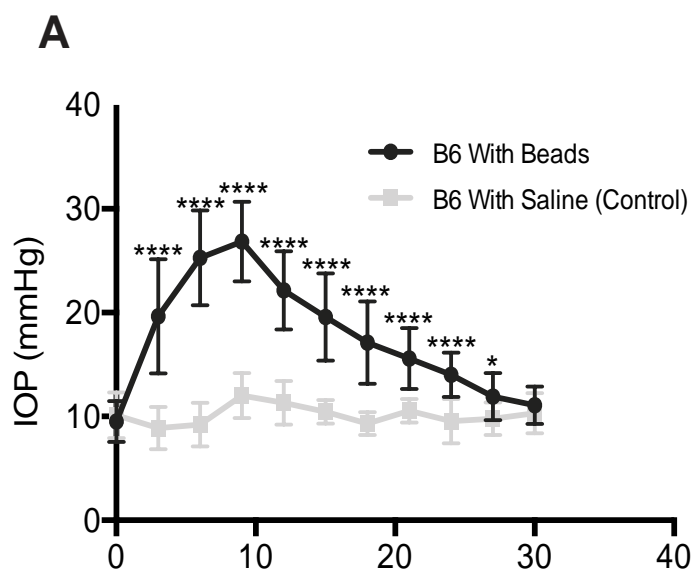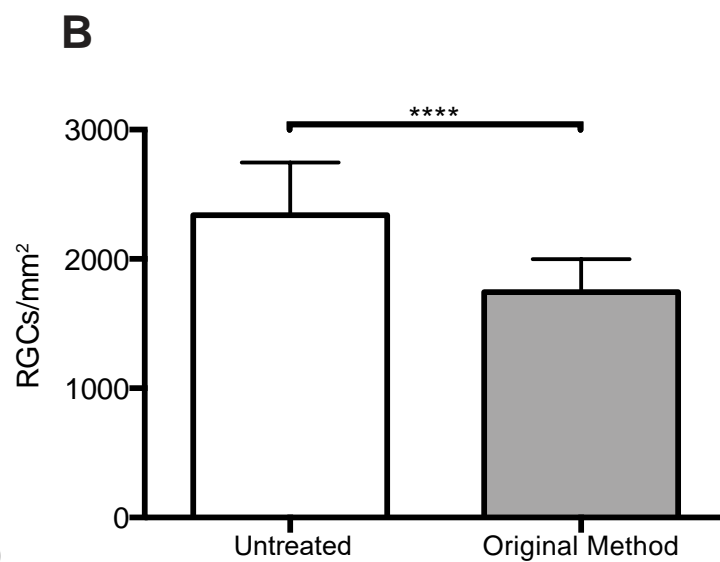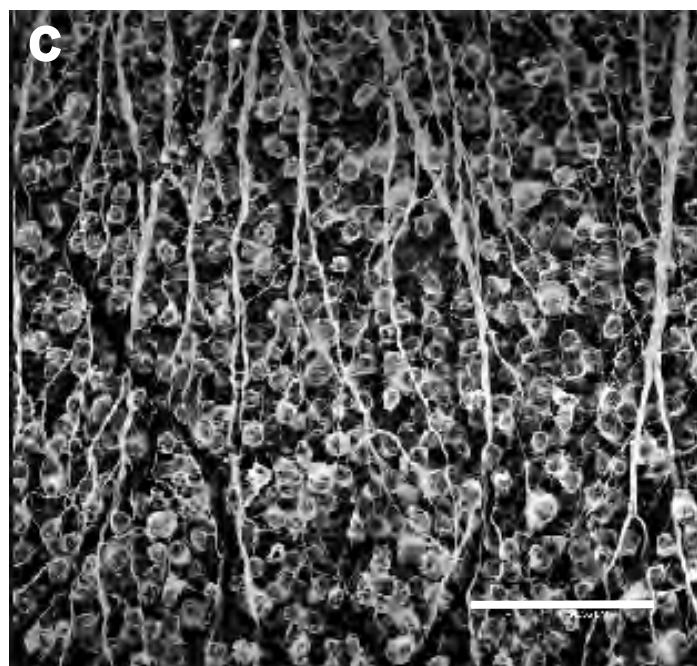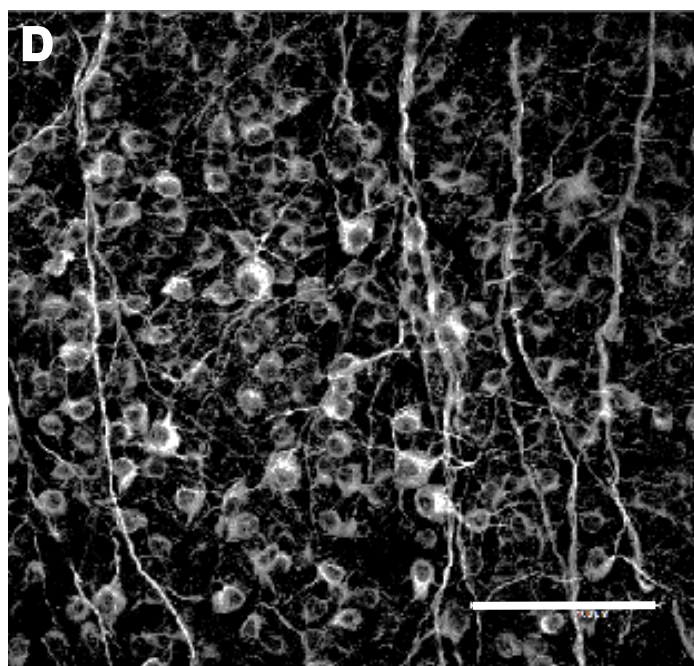

Supplement: S7 Fig — (A) IOP change in B6 mice after injection with 3μl of beads suspended in saline as compared to saline-injected eyes (without beads). Maximum IOP was observed around 7–10 days, with a mean value of 26.83 ± 3.84 mmHg in the B6 mice with beads group. (B) Quantification of RGC density using Tuj-1 stain in untreated (Untreated) and beads-injected (Original Method) B6 mice at 30 days post-treatment. There was a significant reduction in the number of RGCs in the original method group compared to the normal,untreated eye group. Nevertheless, RGC damage was overall minimal. (C) RGC immunostaining with Tuj1 from a normal, untreated B6 mouse. (D) RGC immunostaining with Tuj1 from a beads-injected B6 mouse at 30 days post-injection. Scale bar = 50 μm. (PDF) [file pone.0208713.s007.pdf]

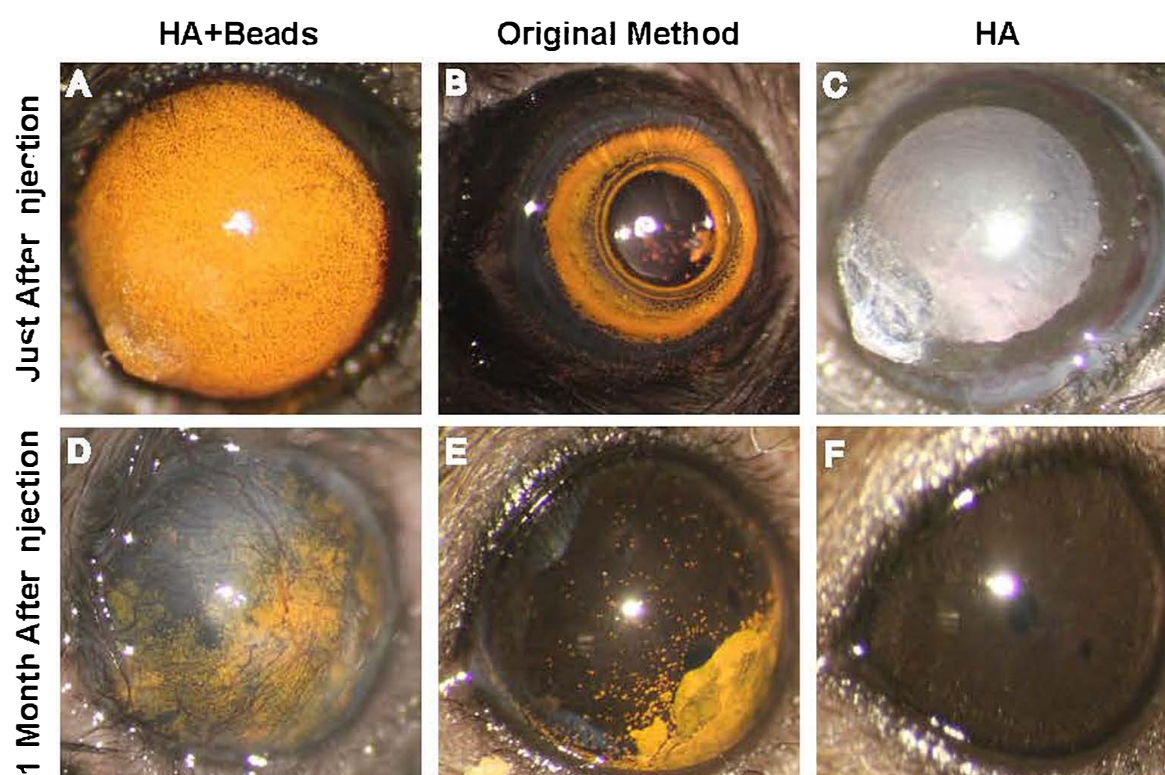

S8 Fig

Supplement: S8 Fig — (A-C) Anterior segment photographs just after injection in each experimental group. (D) Neovascularization was identified in the deep cornea in the hyaluronic acid (HA) + Beads group. (E) Beads were identified inferonasally in the original method group. (F) Anterior segment photographs in the HA group were essentially normal. (PDF) [file pone.0208713.s008.pdf]
