# Supplementary material for: Mouse model of ocular hypertension with retinal ganglion cell degeneration
Source: PLoS One. 2019 Jan 14;14(1):e0208713. doi: 10.1371/journal.pone.0208713 (PMC6331128; doi:10.1371/journal.pone.0208713)
Supplement: S1 Table — In the hyaluronic acid (HA) + Beads group, suspected central retinal vein occlusion (CRVO) occurred in 27% of eyes (3/11 eyes), even though suspected CRVO was not observed after induction of ocular hypertension (OH) in the other experimental groups. (DOCX) [file pone.0208713.s009.docx]

|  | Complicated susupected CRVO after inducing OH |
| --- | --- |
| HA Alone(n=8) | 0% |
| Original Method (n=7) | 0% |
| HA+Beads (n=11) | 27% (3/11 eyes) |

S1 Table. Prevalence of suspected central retinal vein occlusion (CRVO) in each experimental group. In the hyaluronic acid (HA) + Beads group, suspected central retinal vein occlusion (CRVO) occurred in 27% of eyes (3/11 eyes), even though suspected CRVO was not observed after induction of ocular hypertension (OH) in the other experimental groups.
